# Hemicranial postural headache as a first symptom of a spontaneous carotid cavernous fistula: A case report

**DOI:** 10.1097/MD.0000000000031088

**Published:** 2022-10-14

**Authors:** Li Liu, Yushi Zhong, Bin Wu, Xianbi Tang, Ziwei Yi, Chuzheng Pan

**Affiliations:** a Department of Neurology, The First People’s Hospital of Huaihua of University of South China, Huaihua, PR China; b The Advanced Stroke Center of China, Huaihua, PR China; c The Forth People’s Hospital of Huaihua, Huaihua, PR China.

**Keywords:** carotid cavernous fistula, digital subtraction angiography, epistaxis, postural headache

## Abstract

Spontaneous carotid cavernous fistula (CCF) is rare, and the expression of headache caused by it can be variable.

**Patient concerns:**

A case of a man hospitalized for high-intensity hemicranial headache which was aggravated by lying down and relieved when standing or sitting. The pain was of a pulsating character, localized on the right, behind the eye, followed by nausea and vomiting. He gradually appeared with ophthalmoplegia, decreased visual acuity and epistaxis.

**Diagnosis:**

Digital subtraction angiogram (DSA) showed a pseudoaneurysm arising from the internal carotid artery (ICA) that projected anteriorly and medially into the sphenoid sinus with occluded fistula.

**Interventions:**

The pseudoaneurysm was successfully treated with covered stent.

**Outcomes:**

The patient was then followed up clinically at the outpatient and seen in the outpatient clinic with no further episodes of nasal bleeding or new neurologic deficit. The vision loss and ophthalmoparesis were unchanged.

**Lessons:**

Hemicranial postural headache may be the first and characteristic sign of spontaneous CCF.

## 1. Introduction

A carotid cavernous fistula (CCF) is an abnormal communication between the internal carotid artery (ICA) and the cavernous sinus as a result of trauma or ruptured of an antecedent carotid segment aneurysm.^[1]^ Spontaneous CCF accounts for approximately 30% of all CCF^[2]^ and may be caused by ruptured aneurysm, atherosclerosis, or blood vessel inflammation.^[2]^ The clinical presentation of spontaneous CCF includes proptosis, chemosis, headache, diplopia with unilateral ophthalmoparesis, cranial bruit and decreased visual acuity.^[2]^

The mixing of venous and arterial blood leads to high-flow, raised intracranial pressure and arterial steal which might cause the headache. The forms of headache are variable in CCF^[3,4]^

We reported a patient with hemicranial postural headache as a first symptom of a spontaneous CCF.

## 2. Case report

**Figure 1.**
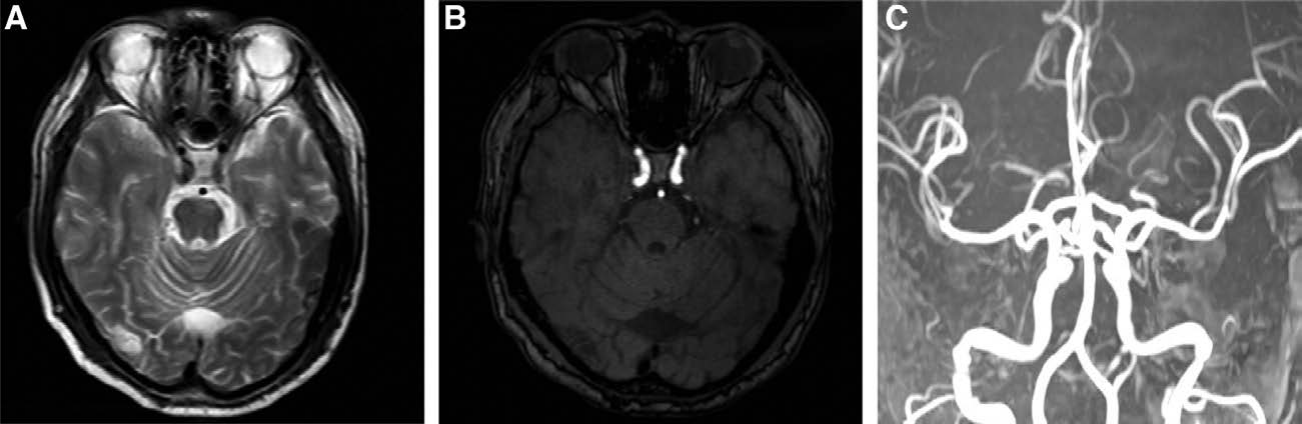
MRI (A) and MRA (B and C) examination with no positive results. MRA = magnetic resonance angiography, MRI = magnetic resource imaging.

**Figure 2.**
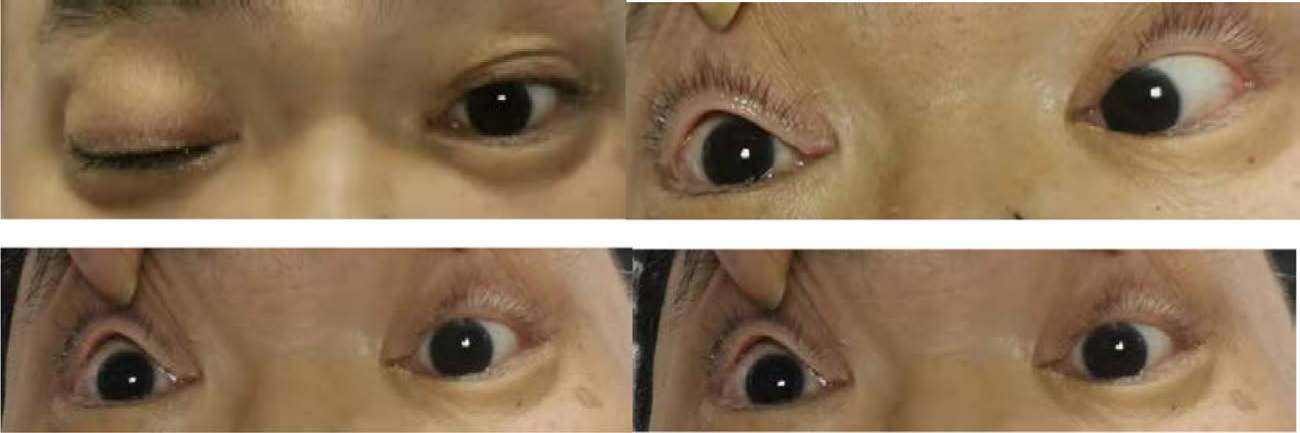
Eye movements demonstrated right oculomotor, abducens, trochlear and left abducens nerve palsy.

A 53-year-old man presented to the hospital with an intensive hemicranial headache without history of traumatic injury. He complained of a sudden headache occurring 2 weeks previously which was aggravated by lying down and relieved when standing or sitting. Since the headache persisted, he visited a local hospital for computed tomography (CT), magnetic resource imaging (MRI) and magnetic resonance angiography (MRA) examination with no positive results (Fig. 1). After 1 week later, the patient exhibited complete right oculomotor, abducens, trochlear and left abducens nerve palsy gradually (Fig. 2). Lumbar puncture was conducted. Opening pressure was normal (16 cm H_2_O). The levels of cerebrospinal fluid (CSF) protein (1081.0 mg/L) were high (range 150‐450 mg/L). However, other CSF profiles were normal. IVIG and symptom-based treatments were given to him at that time without effect. He was admitted to our hospital due to torrential nasal bleeds that stopped spontaneously the first time, but required nasal packing the next time it occurred a few days later.

**Figure 3.**
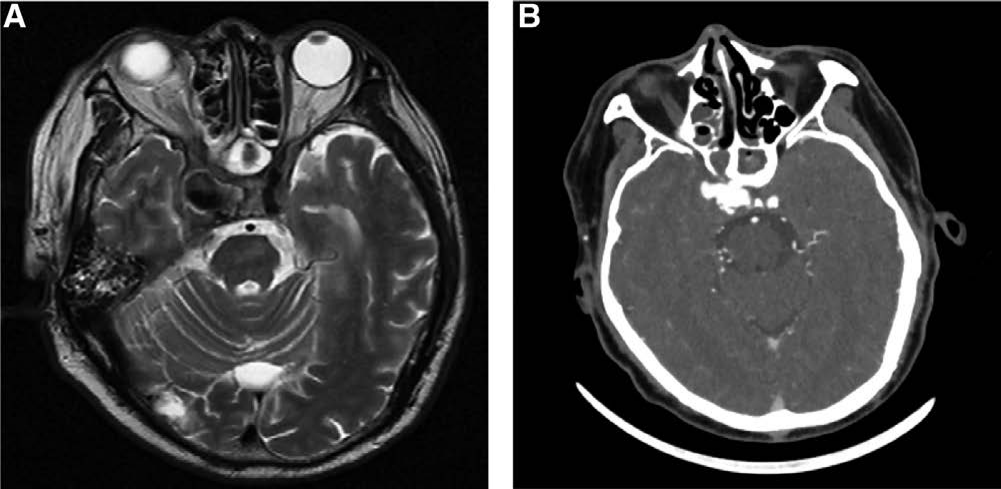
T2-weighted (A) MRI and CT angiogram (B) showing right internal carotid pseudoaneurysm measuring 20 × 15 mm. CT = computed tomography, MRI = magnetic resource imaging.

The reexamination of MRI demonstrated the presence of a large arterial out-pouching arising from the cavernous part of the right ICA protruding through a defect at the right superior-lateral aspect of the sphenoid sinus representing pseudoaneurysm, with its tip projecting well into the right side of the sphenoid sinus (Fig. 3). He was shifted to the angiographic suite for covered stent.

**Figure 4.**
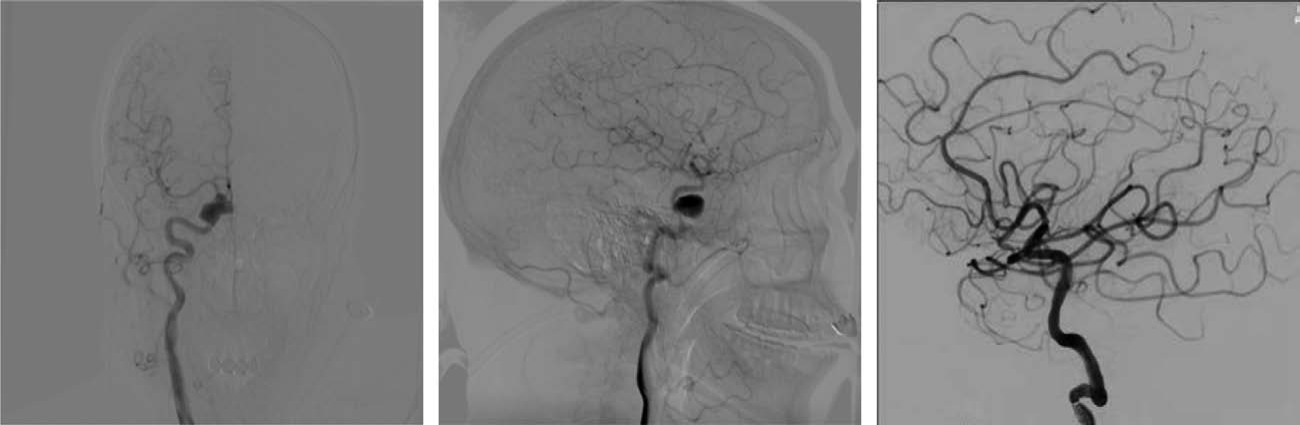
DSA showing right internal carotid pseudoaneurysm and a covered stent remained in stable position across the pseudoaneurysm neck. DSA = digital subtraction angiography.

**Figure 5.**
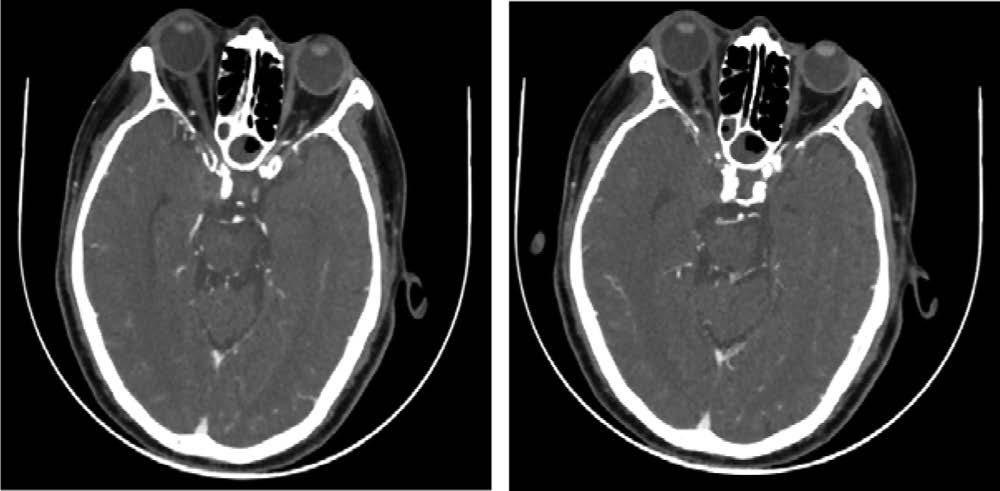
Postoperative CT angiogram showing the disappearance of pseudoaneurysm. CT = computed tomography.

A right ICA cerebral angiogram was performed using the standard projections, as well as oblique projections, and showed a pseudoaneurysm arising from the ICA that projected anteriorly and medially into the sphenoid sinus. All procedures were performed under general anesthesia, after positioning a 5F Navien (Covidien, Mansfield, MA, USA) guiding catheter in the ICA, and a microguidewire (Synchro-2, Boston Scientific, Watertown, MA, USA) was then navigated into a distal branch of the middle cerebral artery. With roadmap guidance, the covered stent was navigated over the microguidewire, and then proceeded to bridge the orifice of the fistula. Angiography was performed immediately after balloon deflation to confirm the correct placement of the stent and satisfactory occlusion of the pseudoaneurysm (Fig. 4). He tolerated the procedure and was extubated upon completion. There were no immediate complications. The postoperative CT angiogram showed the disappearance of pseudoaneurysm (Fig. 5). He was then followed up clinically at the outpatient and seen in the outpatient clinic with no further episodes of nasal bleeding or new neurologic deficit. The vision loss and ophthalmoparesis were unchanged.

## 3. Discussion

Differential diagnosis of hemicranial pain involves many primary and secondary headaches and painful cranial neuropathies. As unilateral pain may be a characteristic of primary headaches, secondary headaches and painful cranial neuropathies, whose clinical presentations often overlap, differential diagnostic dilemmas are often encountered in clinical practice in determining the cause of a hemicranial headache.^[5]^

The case, typically as in other cases, presented with headache and ophthalmoparesis. However, it is unusual in terms of the bilateral eye movement disorder which might misdiagnose as Guillain-Barré syndrome especially with elevated CSF protein and negative MRI. Bilateral ophthalmoparesis from non-traumatic CCF are rare especially without ocular or orbital signs.^[6]^ Since the sixth nerve is in close proximity to the ICA in the cavernous sinus, it is vulnerable when CCF develops. The mechanism is thought to involve direct compression or vascular steal with cranial ischemia.^[7]^

Barrow et al classified CCF into 4 types.^[8]^ Type A are fistulas between the ICA or its branches with the cavernous sinus characterized by rapid and high flow, and are divided into two subtypes: A1, which are most traumatic in origin, and A2, which are the result of rupture of the ICA aneurysm in the cavernous sinus. Type B is the dural shunt of the meningeal branches of the external carotid artery with a cavernous sinus. Type C is a dural shunt of the meningeal branches of the external carotid and with a cavernous sinus, and type D is a combination of types B and C. Our patient might have CCF type A2 with occluded fistula. What is unusual and differentiates our case from other published case reports with CCF is that our patient had a postural headache which was aggravated by lying down and relieved when standing or sitting. The mechanism of the symptoms might due to the increased venous hypertension as the venous return reduced when patient lying down.

The absence of abnormalities on noninvasive imaging studies does not exclude the diagnosis of CCF. If there is a high degree of clinical suspicion is consistent with the presence of CCF, the patient should be referred for diagnostic catheter cerebral angiography. Timely diagnosis and adequate treatment play a key role in the prognosis, as they can prevent the complications that CCF can cause, the most serious of which are blindness, intracranial hemorrhage and venous infarctions.^[2]^ However, some CCF patients only present with headache as in our case without other symptoms that would indicate a fistula in the early time. Choosing the patients with specific headache undergoing digital subtraction angiography (DSA) is important to avoid the delay in the diagnosis of CCF.

In our case, we recommend that additional test, particularly DSA, should be performed in patients with a sudden hemicranial postural headache.

## 4. Conclusion

Hemicranial postural headache may be the first and characteristic sign of spontaneous CCF, and in such cases, a fistula should be suspected, and detailed diagnostic tests should be performed in order to diagnose and prevent serious complications of CCF.

## Declaration

This case was reported or written according to ethical committee of the First People's Hospital of Huaihua criteria for reporting or writing case reports. The patient and relatives were informed about our intension to involve him in a case study and they agreed to partake in the study.

## Author contributions

**Conceptualization:** Bin Wu, Chuzheng Pan.

**Investigation:** Li Liu, Yushi Zhong, Bin Wu.

**Resources:** Li Liu, Yushi Zhong, Bin Wu, Xianbi Tang, Chuzheng Pan.

**Writing – original draft:** Li Liu, Xianbi Tang, Chuzheng Pan.

**Writing – review & editing:** Ziwei Yi, Chuzheng Pan.
